# Neuroprotective Activity of a Non-Covalent Imatinib+TP10 Conjugate in HT-22 Neuronal Cells In Vitro

**DOI:** 10.3390/pharmaceutics16060778

**Published:** 2024-06-07

**Authors:** Izabela Rusiecka, Iwona Gągało, Ivan Kocić

**Affiliations:** Department of Pharmacology, Medical University of Gdańsk, Dębowa 23, 80-204 Gdańsk, Poland

**Keywords:** Parkinson’s disease, cell-penetrating peptides, transportan 10, TP10, imatinib, CPP

## Abstract

This study evaluated the probable relevance of a non-covalent conjugate of imatinib with TP10 in the context of a neuroprotective effect in Parkinson’s disease. Through the inhibition of c-Abl, which is a non-receptor tyrosine kinase and an indicator of oxidative stress, imatinib has shown promise in preclinical animal models of this disease. The poor distribution of imatinib within the brain tissue triggered experiments in which a conjugate was obtained by mixing the drug with TP10, which is known for exhibiting high translocation activity across the cell membrane. The conjugate was tested on the HT-22 cell line with respect to its impact on MPP^+^-induced oxidative stress, apoptosis, necrosis, cytotoxicity, and mortality. Additionally, it was checked whether the conjugate activated the ABCB1 protein. The experiments indicated that imatinib+PEG_4_+TP10 reduced the post-MPP^+^ oxidative stress, apoptosis, and mortality, and these effects were more prominent than those obtained after the exposition of the HT-22 cells to imatinib alone. Its cytotoxicity was similar to that of imatinib itself. In contrast to imatinib, the conjugate did not activate the ABCB1 protein. These favorable qualities of imatinib+PEG_4_+TP10 make it a potential candidate for further in vivo research, which would confirm its neuroprotective action in PD-affected brains.

## 1. Introduction

Parkinson’s disease (PD) is a common late-onset progressive neurodegenerative disorder that is characterized by severe motor impairment and a non-motor deficit. The current therapeutic options are all aimed at symptomatic relief, resulting mainly from the supplementation of dopamine (DA) within the nigrostriatal system. This approach allows patients to overcome most of their motor symptoms for a considerable period of time; however, non-motor functions that adversely impact the patient’s quality of life remain largely obstinate to the available pharmacological interventions. So far, no therapeutic modality can halt the progression of the illness that is tightly linked to the ongoing neurodegenerative process in the brain areas affected during PD. Hence, there is a great demand for a new effective pathogenesis-relevant therapy for PD that can modify the course of the illness and simultaneously restore all the compromised functions.

Research on the pathophysiological process of PD itself brought about the identification of novel therapeutic targets for neuroprotection [1]. One of them addresses the c-Abl non-receptor tyrosine kinase (TK), whose involvement in the pathogenesis of this illness has been widely observed. Its upregulation (demonstrated in post-mortem human brains and in animal models of PD) [2], due to mitochondrial dysfunction and the resulting oxidative stress, triggers multiple pathogenic signals, leading to the inactivation of parkin (the loss of autoubiquitination and the accumulation of pathogenic parkin substrates); the activation of p38α (a member of the MAP kinase family); microglial NLRP3 inflammasome neuroinflammation; and α-synuclein phosphorylation (aggregation of α-synuclein). The first three pathologies merge into cellular death, and the last one is most probably implicated in neuronal toxicity [2,3].

The pharmacological inhibition of aberrant c-Abl activity has been successfully used in oncology [4]. This strategy has also been validated as a potential disease-modifying PD therapy. As far as this issue is concerned, diverse tyrosine kinases inhibitors (TKIs), e.g., imatinib (Im), nilotinib, and bafetinib [5,6,7,8], have been reported to increase brain DA and improve motor behavior and cognitive skills [9]. It is worth stressing that, by reversing the principal pathologies induced by the over-activation of c-Abl, these drugs have the capacity to restore the function of parkin and protect the neurons against α-synuclein toxicity in the autophagy-dependent pathway [10]. 

Generally, the TKIs exploited in PD research comprise small-molecular-weight lipophilic compounds. The prototype of them is imatinib mesylate (Gleevec), which is known for its inhibition of non-receptor c-Abl kinase. In addition, the drug inhibits c-kit, PDGFR, and other receptor TKs, e.g., Flt-3 and c-fms, which are critical components of neuroinflammatory processes [11].

As a result of sharing the physicochemical properties of the TKI group, imatinib should gain easy access to all compartments of the body. However, this does not affect its distribution within the brain. The unsatisfactory concentrations that usually occur mainly result from the fact that the drug, as a substrate for ABC-family transporters, is actively pumped out of brain cells [11]. This pharmacokinetic quality of imatinib may also be ascribed to many other TKIs [11]. 

The versatile and limited CNS exposure to these compounds does not exclude their presence in the brain under pathological conditions in which the blood–brain barrier (BBB) loses its integrity. These pathological conditions comprise versatile central disorders, including PD, for which TKIs have shown therapeutic potential in numerous animal models [9] and in preclinical studies [12,13].

There are diverse procedures aimed at the facilitation of therapeutic molecule transport into the cell interior of eukaryotes. One of them is the use of a drug conjugate with a cell-q1-penetrating peptide (CPP) [14]. These peptides constitute a class of oligopeptides rich in basic amino acids and characterized by exceptional translocation properties across cell membranes, including those that form vital biological barriers without significant interference with their integrity. These compounds originate from a wide variety of sources and usually carry a positive net charge. A well-known CPP is TP10 (transportan 10), which is a chimeric primary amphipathic peptide with a high cell-penetrating capacity resulting from the destabilization or disruption of the cellular membrane [14,15,16]. 

Without a doubt, CPPs, as pharmacological modifiers, have proven their utility for the delivery of otherwise-impermeable macromolecular therapeutic cargoes into the intracellular compartment. Various strategies have been used for associating the peptide with the therapeutic molecule. The main options involve covalent or non-covalent interactions [14,15].

There is evidence that many diseases could potentially benefit from such a delivery system, and such systems have been tested most frequently in cancers [15]. Also, neurological disorders such as PD or Alzheimer’s disease have recently become the targets of this kind of research [16].

Therefore, the purpose of the present study was to investigate the therapeutic activity of a non-covalent construct of TP10 with imatinib in PD. The experiments were performed on a hippocampal neuronal HT-22 cell line that was used as a model for this neurological disorder [17]. The construct was evaluated with regard to its impact on the MPP^+^-induced oxidative stress, neurotoxicity, and activity of the ABCB1 protein (glycoprotein P; P-gp).

## 2. Materials and Methods

### 2.1. Cell Line and Culture Conditions

Mouse hippocampal neuronal HT-22 cells (widely used as a model for PD) obtained from ATCC (Manassas, VA, USA) were cultured as a monolayer in a growth medium containing DMEM (Merck, Darmstadt, Germany), 10% FBS (Merck, Darmstadt, Germany), 4 mM L-glutamine (Merck, Darmstadt, Germany), and 1% PEN/STREP (Merck, Darmstadt, Germany). The cells were maintained at 37 °C in a humidified atmosphere of air (95%) and CO_2_ (5%). They were regularly split and subcultured up to 70–90% of confluence.

### 2.2. Peptides and Constructs

The compounds, i.e., TP10 (AGYLLGKINLKALAALAKKIL-NH2) and TAT (GRKKRRQRRRPQ-NH2), were synthetized to an individual order by Pepmic Co., Ltd. (Beijing, China). The full HPLC analyses of the compounds in question are provided in Figures S1 and S2. PEG_4_ (the linker), imatinib, and the rest of the utilized reagents (unless otherwise noted) were obtained from Merck (Darmstadt, Germany). The non-covalent conjugates of TP10, i.e., imatinib+TP10 and imatinib+PEG_4_+TP10, were prepared by mixing the stock solution of TP10 with imatinib or with both PEG_4_ and imatinib in a ratio of 1:1 or 1:1:1, respectively. The same procedure was used for obtaining the non-covalent conjugates of TAT. 

### 2.3. Induction of Oxidative Stress

Oxidative stress was induced by MPP^+^ (Merck, Darmstadt, Germany), a toxic metabolite of the neurotoxin MPTP, which causes symptoms of PD in animal models by selectively damaging dopaminergic neurons in the substantia nigra. This metabolite is taken up by the DA transporter into dopaminergic neurons, where it exerts its neurotoxic action on mitochondria by affecting complex I of the respiratory chain. 

An amount of 1 mM of MPP^+^ was dissolved in ultrapure sterile water and freshly prepared for each experiment. This concentration (according to experiments by Zeng et al.) induced the significant production of reactive oxygen species (ROS) [18], which are an indicator of the oxidative stress severity. 

### 2.4. Oxidative Stress Assay

The ROS-Glo™ H_2_O_2_ assay operates by detecting and measuring H_2_O_2_ using a H_2_O_2_ substrate, which directly reacts with H_2_O_2_ present in the sample to produce a luciferin precursor. The addition of the ROS-Glo detection solution converts the precursor to luciferin and provides luciferase to produce a light signal (measured as luminescence) proportional to the amount of H_2_O_2_. This procedure enables the precise determination of the H_2_O_2_ concentration in a sample.

The oxidative stress assay was carried out according to the original protocol provided by Promega (Madison, WI, USA). Briefly, 100 μL of a medium containing HT-22 cells was seeded in a 96-well white luminometer plate (Corning, Corning, NY, USA) with a density of 10^5^ cells/well. The cells were allowed to grow for 24 h. Next, they were exposed to 1 mM of MPP^+^ for the following 24 h. After this period of time, the H_2_O_2_ substrate (25 μM in a volume of 10 μL) and the tested compounds (1 μM, 2.5 μM, and 5 μM) were added. The latter compounds included the following: TP10, PEG_4_, imatinib, imatinib+TP10, imatinib+PEG_4_+TP10, TAT, imatinib+TAT, and imatinib+PEG_4_+TAT. As a positive control, 20 μL of the H_2_O_2_ substrate was used. Subsequently, the samples were incubated (4 h period), and following that, they were exposed to the ROS-Glo detection solution for 20 min. Finally, the luminescence was measured with the use of a GloMax^®^ Explorer microplate reader (Promega, Madison, WI, USA). The results are presented as the % of ROS production observed in the cells treated with MPP^+^ and the tested compounds in comparison to the percentage obtained after exposure to MPP^+^ alone (100%). 

### 2.5. Neuroprotective Activity Assay 

An exponent of neuroprotective action is a reduction in neuronal apoptosis and/or necrosis. These processes were estimated by the RealTime-Glo™ Annexin V apoptosis and necrosis assay (Promega, Madison, WI, USA). This assay is a live-cell (non-lytic) real-time (kinetic) assay that measures the intensity of the luminescence resulting from the binding of annexin V (subunit of luciferase) to phosphatidylserine (PS). This phospholipid is exposed on the outer leaflet of the cell membrane during the apoptotic process. Furthermore, this assay detects the membrane condition: if there is a loss of membrane integrity, the DNA-binding dye will enter the cell and generate a fluorescent signal. Therefore, the kind of signal registered after treatment may reflect an apoptotic or necrotic form of cell death. 

The RealTime-Glo™ Annexin V apoptosis and necrosis detection reagent was prepared (as indicated in the technical protocol) just before starting the experimental procedure. It consisted of annexin V fusion proteins (SmBiT and LgBiT, which are complementary subunits of luciferase–NanoBiT), CaCl_2_, a time-released luciferase substrate, and a cell-impermeant profluorescent DNA dye. 

At the beginning, 100 μL of a medium containing HT-22 cells was seeded in a 96-well white luminometer plate (Corning, Corning, NY, USA) with a density of 10^5^ cells/well. The cells were allowed to grow for 24 h. After this period of time, MPP^+^ (1 mM), imatinib, imatinib+TP10, imatinib+PEG_4_+TP10, and two detection reagents were added to an appropriate well. The chosen concentration of the tested compounds was 2.5 μM, as that was the optimal ratio between activity and toxicity. The control group constituted the cells treated with MPP^+^ alone. The measurements were performed in the kinetic mode according to scheduled intervals, i.e., after 0, 4, 8, 12, and 16 h. During the intervals between the measurements, the assay plates with lids were placed in a humidified tissue culture incubator. Subsequently, the intensity of luminescence and fluorescence (485 nmEx/525-530 nmEm) was measured with the use of a GloMax^®^ Explorer microplate reader (Promega, Madison, WI, USA). 

### 2.6. Cell Survival Assay

The procedure for preparing fluorescent samples was as follows. Coverslips (∅20 mm, Carl Roth, Karlsruhe, Germany) were washed with concentrated HCl, rinsed with 70% ethanol, and placed at the bottom of a 12-well plate. Cells were seeded at a density of 10^5^ per ∅22 mm well (12-well plate) and allowed to adhere for 24 h. Following this, the cells were washed three times with PBS; then, 1 mM of MPP^+^ (Merck, Darmstadt, Germany) in 200 μL of DMEM was added to each well and the plates were incubated for 1 h. Subsequently, imatinib and imatinib+PEG_4_+TP10 were added to the wells at a concentration of 2.5 μM each, and the samples were incubated for a further 24 h. The next step involved staining the cell nuclei by adding 1 μM of the blue dye HO3342 (Hoechst 3342, Merck, Darmstadt, Germany) to the samples. The cells were then fixed in a 4% formaldehyde solution for 15 min (diluted from a 37% stock solution) and placed upside down on a Superfrost glass slide (Carl Roth, Karlsruhe, Germany). A drop of Vectashield mounting medium (Vector Laboratories, Burlingame, CA, USA) was placed between each glass slide and coverslip. The edges of the coverslips were sealed with nail polish. Visualization was performed using a fluorescence microscope (Delta Optical IB-100, Delta Optical, Mińsk Mazowiecki, Poland) at an emission intensity of 352 nm.

### 2.7. Activation of the Cellular ABCB1 Protein 

Western blotting was applied for the detection of the MDR system component, i.e., ABCB1. For this purpose, anti-P-gp monoclonal antibodies (P7965; Merck, Darmstadt, Germany) directed to a highly conserved epitope of human ABCB1 were used. 

The HT-22 cells were seeded in a 150 mm dish (Merck, Darmstadt, Germany) and cultured at 37 °C in an atmosphere of 95% air/5% CO_2_ until obtaining confluence at the level of 70%. 

Again, oxidative stress was induced by 1 mM of MPP^+^. Next, the cells were exposed to the treatment, i.e., 2.5 μM of imatinib, TP10, or imatinib+PEG_4_+TP10 was added to each respective well. After 24 h, the cells were scraped in ice-cold PBS/protease inhibitor cocktail, lysed (0.1% Triton X-100 in PBS), spun down (at 14,000 rpm for 5 min), and finally subjected to a Western blot analysis.

The total protein lysate (10 μg) was loaded onto each lane and electrophoresis was performed on a 6% polyacrylamide gel, as described by Laemmli (1970) [19]. A wide-range molecular mass protein marker of 24–180 kDa (Merck, Darmstadt, Germany) was used as a reference. After the transfer onto a PVDF membrane (Merck Millipore, Billerica, MA, USA) and its blocking with 5% non-fat dry milk (Mlekovita, Wysokie Mazowieckie, Poland) in PBS at 4 °C overnight, the blots were incubated with anti-P-gp in PBS (1:100) at 4 °C. Secondary antibodies conjugated to horseradish peroxidase (Santa Cruz Biotechnology, Santa Cruz, CA, USA; 1:3000) and a BM Chemiluminescence Western Blotting Kit (Roche Diagnostic, Penzberg, Germany) were used to develop images on an autoradiography film (Kodak X-Omat AR, Rochester, NY, USA).

### 2.8. Cytotoxicity Determination of Imatinib, TP10, and Imatinib+PEG_4_+TP10 

To determine the cytotoxicity of the compounds in question, two assays were used: MTT for the determination of cytotoxicity (Section 2.8.1) and IC50 (Section 2.8.2) and CellTox™ Green Cytotoxicity (Section 2.8.3) for an estimation of cellular death in the cell culture. 

#### 2.8.1. Colorimetric Cytotoxicity Assay

The MTT assay [3-(4,5-dimethylthiazol-2-yl)-2,5-diphenyl-2H-tetrazolium bromide] included the following experimental groups: imatinib, TP10, and imatinib+PEG_4_+TP10. These compounds were tested at concentrations from 0.1 to 50 μM on the HT-22 cell line. A control group without any tested compounds was also included.

The MTT assay (Merck, Darmstadt, Germany)followed a standardized protocol, as detailed in a previous publication [15]. Concisely, MTT (5 mg/mL) was dissolved in PBS, sterilized using a 0.22 μm Millipore^®^ filter (Merck Millipore, Billerica, MA, USA), and stored at 4 °C. The HT-22 cells were seeded at a density of 10⁴ cells per well in a 96-well plate (Corning, Corning, NY, USA) with 100 μL of culture medium and allowed to grow for 24 h before the addition of imatinib, TP10, or imatinib+PEG_4_+TP10. After an additional 24 h, the cells were washed twice with PBS, and 100 μL of 0.5 mg/mL MTT in a serum-free medium was added to each well. The incubation was carried out at 37 °C for a further 3 h to allow for MTT metabolism. The resulting formazan crystals were dissolved in 100 μL of acidified isopropanol (Merck, Darmstadt, Germany), and the absorbance was measured at 570 nm using a GloMax^®^ Explorer microplate reader (Promega, Madison, WI, USA).

The results are expressed as a percentage of the control value, which is relative to the untreated cells (100%).

#### 2.8.2. Determination of IC50

The raw data obtained from the experiments of the MTT assay (Section 2.8.1) were exported to the GraphPad 9.0 software for subsequent IC50 calculations.

#### 2.8.3. Fluorescence Cytotoxicity Assay

To determine the cytotoxicity of the tested compounds, the CellTox™ Green Cytotoxicity Assay (Promega, Madison, WI, USA) was used. This test measures changes in the membrane integrity of the cells. The assay system uses a proprietary asymmetric cyanine dye, which is excluded from viable cells, but preferentially stains the dead cells’ DNA. When the dye binds DNA in compromised cells, its fluorescent properties are substantially enhanced. Viable cells produce no appreciable increases in fluorescence. Therefore, the fluorescent signal produced by the dye bound to the dead-cell DNA is proportional to cytotoxicity. 

In this experiment, HT-22 cells were seeded in a 96-well black plate (Corning, Corning, NY, USA) at a density of 10^5^ cells/well and incubated for 24 h. Next, a mixture of CellTox™ Green Dye and cell medium (1:1000) was added to each well. Subsequently, the cells were treated with imatinib, TP10, imatinib+TP10, or imatinib+PEG_4_+TP10 at a concentration of 2.5 μM. Wells with untreated cells or without cells were used as control or background wells, respectively. Cell death was determined by measuring the fluorescence intensity (485/520 nm excitation/emission) with a GloMax^®^ Explorer microplate reader (Promega, Madison, WI, USA) 0, 6, 12, and 24 h after treatment. 

The posttreatment values of fluorescence (expressed in RFU) were calculated with regard to the fluorescence of the control. 

### 2.9. Statistics

The results from the luminescent, fluorescent, and colorimetric assays are presented as the mean of three independent experiments, each performed in triplicate for every concentration. Based on these results, the standard error of measurement (SEM) was calculated. The fluorescence microscopy and Western blotting data were derived from at least three repeated experiments. The data analysis was performed using two tests, the Friedman test and the Kendall concordance test, with the STATISTICA version 13.3 software (StatSoft, Inc., Kraków, Poland, Site License, accessed on 1 October 2023). The results were considered to be statistically significant (*) at *p* < 0.05. The IC50 values were determined using the four-parameter logistic (4PL) method via GraphPad Prism 9.0 (GraphPad Software, Boston, MA, USA), and were validated with the R software, version 4.3.1 (The R Foundation, Indianapolis, IN, USA).

## 3. Results

### 3.1. Oxidative Stress 

**Figure 1:**
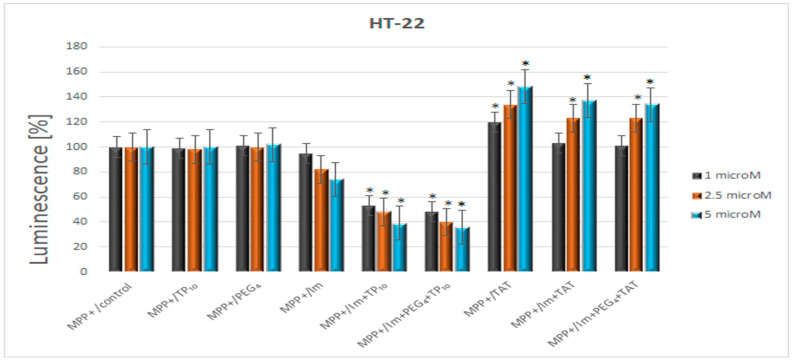
The effect of the Im (imatinib)+PEG_4_+TP10 and Im+PEG_4_+TAT mixtures and their constituents on the MPP^+^-induced oxidative stress. * Statistically significant (*p* < 0.05) as compared to the control.

The effect of the tested compounds on oxidative stress is presented in Figure 1.

Irrespective of the used concentration, TP10, like the linker PEG_4_, did not change the luminescence induced by MPP^+^. Imatinib slightly suppressed oxidative stress, which became mainly visible at the highest concentration (a drop in luminescence by 26%). The inhibitory effect was much more pronounced when the drug was used in the form of conjugates with TP10. Imatinib+TP10 caused a decrease in luminescence in the range of 47–61%. The addition of the linker to the conjugate minimally improved the effect (the luminescence dropped from 52 to 64%). 

The second CPP–TAT enhanced the post-MPP^+^ oxidative stress in a dose-dependent manner. The observed percentage increases in luminescence were as follows: 20, 34, and 48. Its conjugate with imatinib (with the linker or without) intensified the oxidative stress in a way that was comparable to that of TAT.

### 3.2. Neuroprotective Activity Assay 

**Figure 2:**
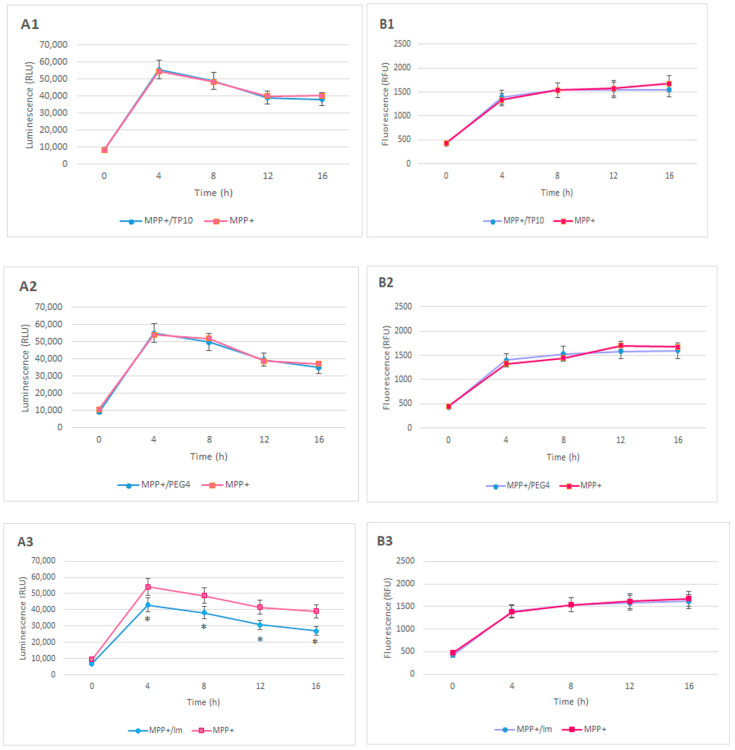

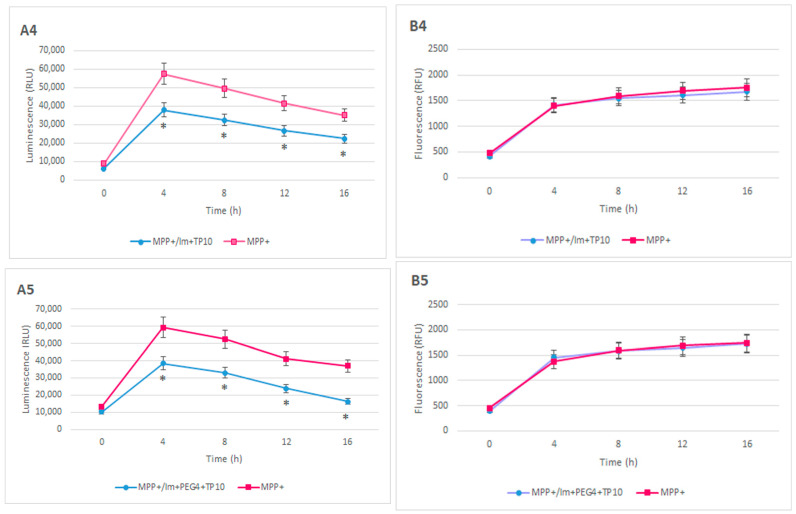
Sequentially repeated measurements of luminescence (**A1**–**A5**) and fluorescence (**B1**–**B5**) in MPP^+^ treated HT-22 cells after administration of the following: (**A1**,**B1**) TP10; (**A2**,**B2**) PEG_4_; (**A3**,**B3**) Im; (**A4**,**B4**) Im+TP10; and (**A5**,**B5**) Im+PEG_4_+TP10. * Statistically significant (*p* < 0.05) as compared to the control.

Changes in post-MPP^+^ luminescence intensity after treatment with TP10, PEG_4_, imatinib, imatinib+TP10, or imatinib+PEG_4_+TP10 (Figure 2(A1–A5)).

The time courses of the MPP^+^-induced luminescence intensity curves presented in Panel A are comparable, considering each experimental set (A1–A5). The maximum luminescence (ca. 55,000 RLU) was observed 4 h after the MPP^+^ treatment. Next, the values of this parameter slowly decreased and reached an intensity of ca. 37,000 RLU at the end of the experimental period (16 h). 

TP10 as well as PEG_4_ caused almost the same changes in the luminescence intensity as MPP^+^. Therefore, the curves reflecting them are overlapping (A1 and A2). 

The imatinib treatment slightly reduced the post-MPP^+^ luminescence intensity. The time course of the curve resembled that of MPP^+^. However, the values at 4 h and 16 h were 43,000 and 27,000 RLU, respectively (A3).

A more evident effect was obtained when imatinib was used in the form of conjugates with TP10 (without the linker—A4; with it—A5). Both compounds produced comparable decreases in the MPP^+^-induced luminescence, although those resulting from imatinib+PEG_4_+TP10 were more intense, especially at the 12th (41%) and 16th (56%) hour of the observation, as compared to those resulting from imatinib+TP10 (more uniform decreases kept at the level of about 36% during the whole experimental period). 

Changes were observed in the post-MPP^+^ fluorescence intensity after treatment with TP10, PEG_4_, imatinib, imatinib+TP10, or imatinib+PEG_4_+TP10 (Figure 2(B1–B5)). 

The MPP^+^-induced fluorescence intensity pattern was similar in each of the experimental sets (B1–B5). The starting values of fluorescence in all cases were comparable and fluctuated between 435 and 476 RFU. An evident increase in this parameter always occurred at 4 h into the experiment, and its level evolved to an average value of 1362 RFU. Next, the intensity of fluorescence was further increased so that its average was 1704 RFU at the end of the experimental period. Generally, the impact of the tested compounds was negligible, and therefore, the time course of the curves obtained after their exposition practically did not change, as compared with that observed after MPP^+^ itself.

### 3.3. Cell Survival Assay

**Figure 3:**
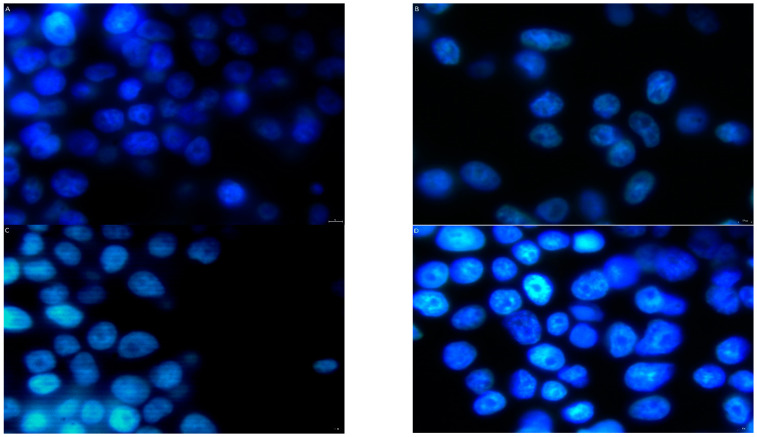
HT-22 cell viability of the following: (**A**) control; (**B**) MPP^+^; (**C**) MPP^+^+Im; and (**D**) MPP^+^+Im+PEG_4_+TP10, visible using fluorescence microscopy (×60 objective).

Figure 3 shows fluorescent images of the HT-22 cells whose nuclei became visible due to the HO3342 blue dye. These images show the number of cells under control conditions (Figure 3A), after exposure to MPP^+^ (Figure 3B), and after exposure to MPP^+^ plus the following tested compounds: imatinib (Figure 3C) or imatinib+PEG_4_+TP10 (Figure 3D). In the control, there was a considerable number of cells (Figure 3A) that constituted a reference for those obtained after the treatment. Figure 3B indicates an evident decrease in the number of HT-22 cells due to MPP^+^ exposition. A cytoprotective effect was observed after the administration of imatinib (Figure 3C) and especially after the administration of imatinib+PEG_4_+TP10 (Figure 3D). The action of the latter compound was really impressive; the number of cells in Figure 3D is comparable to that observed in the control (Figure 3A). 

### 3.4. Activation of MDR System

**Figure 4:**
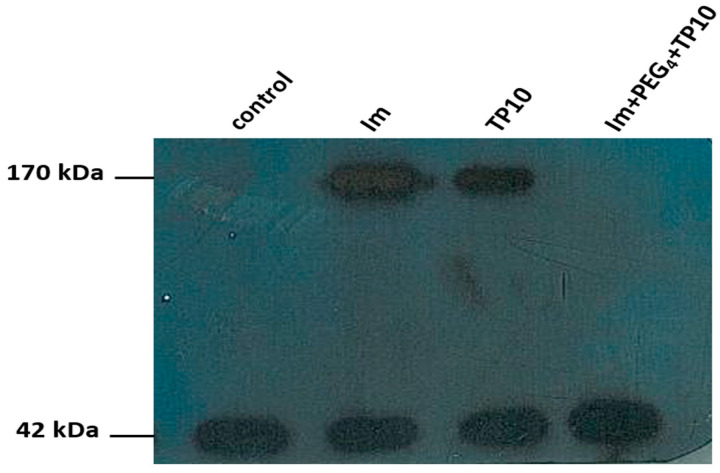
The impact of Im, TP10, and Im+PEG_4_+TP10 on the ABCB1 (170 kDa) protein in MPP^+^ treated HT-22 cell lysates. An actin band of 42 kDa was used as a loading control.

Figure 4 presents changes in the level of the ABCB1 protein induced by the treatment of the compounds in question. Under the control conditions, there were no visible levels of the 170 kDa ABCB1 protein detected. On the other hand, imatinib as well as TP10 evidently activated this protein. Interestingly, there was no activation of the ABCB1 protein due to the incubation with the non-covalent conjugate imatinib+PEG_4_+TP10.

### 3.5. Cytotoxicity of Imatinib, TP10, and Imatinib+PEG_4_+TP10

The cytotoxic effect of the compounds in question was expressed by the cell survival (Section 3.5.1), IC50 values (Section 3.5.2), and cell mortality (Section 3.5.3). 

#### 3.5.1. Colorimetric Cytotoxicity Assay

**Figure 5:**
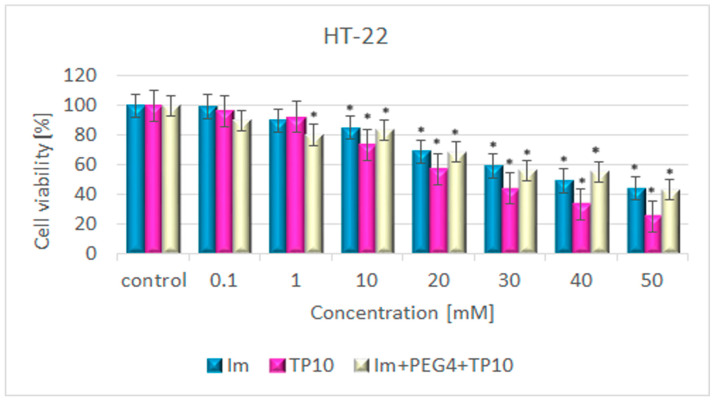
The impact of Im, TP10, and Im+PEG_4_+TP10 on HT-22 cell viability. * Statistically significant (*p* < 0.05) as compared to the control.

The results of the MTT test are presented in Figure 5. All the tested compounds caused gradual decreases in the cell viability as their concentrations increased. Up to 1 μM, the drops in cell viability were comparable and did not exceed 20%. Within a range of concentrations between 10 and 50 µM, the most prominent cytotoxicity, with decreases in cell viability fluctuating from 26 to 75%, was observed in response to TP10 administration. A smaller cytotoxic effect was produced by imatinib and its construct. The maximum inhibition of viability not exceeding 60% was only noted at the highest concentrations of these compounds, i.e., at 50 µM.

#### 3.5.2. IC50 Values

**Figure 6:**
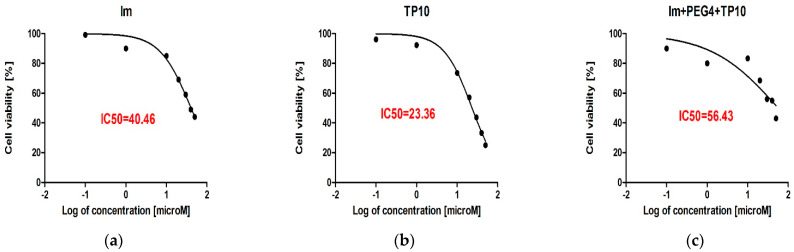
The IC50 values of (**a**) Im; (**b**) TP10; and (**c**) Im+PEG_4_+TP10, calculated by the non-linear four-parametric method.

Figure 6 illustrates the IC50 values calculated for imatinib, TP10, and imatinib+PEG_4_+TP10, which were 40.46 μM, 23.36 μM, and 56.43 μM, respectively. These values provide crucial insight into the inhibitory potency of these compounds against the biological activity (viability) of HT-22 cells. As could be expected, TP10 showed the highest inhibitory potential (IC50 = 23.36 μM). At the same time, those of imatinib and its construct were smaller, i.e., 40.46 μM and 56.43 μM, respectively.

#### 3.5.3. Fluorescence Cytotoxicity Assay

**Figure 7:**
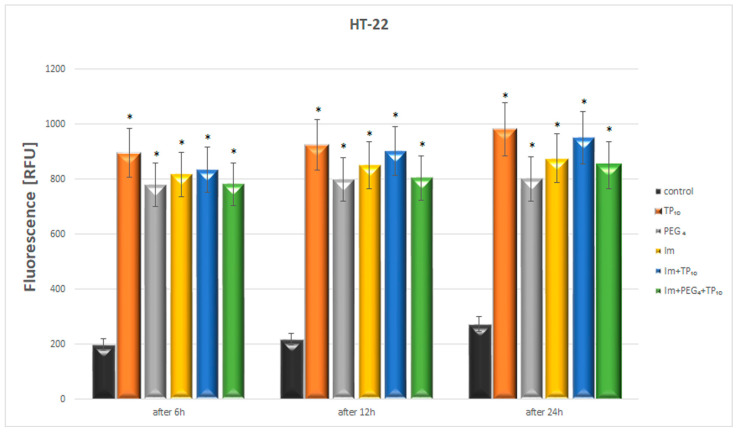
The cytotoxic effect of TP10, PEG_4_, Im, Im+TP10, and Im+PEG_4_+TP10 on HT-22 cells. * Statistically significant (*p* < 0.05) as compared to the control.

As Figure 7 indicates, TP10, PEG_4_, imatinib, imatinib+TP10, and imatinib+PEG_4_+TP10 produced statistically significant increases in the fluorescence observed after 6, 12, and 24 h in comparison to those of the control. The most evident changes ranging from 895 to 981 RFU were noticed after the administration of TP10. Imatinib and imatinib+PEG_4_+TP10 (with a linker) slightly reduced the TP10-induced increases in the fluorescence (in the range of fluorescence from 781 to 851 RFU).

## 4. Discussion

As the present study indicates, the tested non-covalent conjugate of imatinib and TP10 possesses promising qualities that could be noteworthy in the context of PD treatment. These qualities comprise a reduction in the post-MPP^+^ oxidative stress and apoptosis, resulting in the enhanced survival of HT-22 neurocytes. It is worth stressing that this activity of the conjugate was more pronounced than that of imatinib. Moreover, the conjugate evaded the MDR system, which is known to be activated by the latter compound. 

The improved pharmacological activity of the conjugate obtained in this study will be further analyzed in light of the pre-clinical data concerning the action of imatinib on the neurodegenerative processes caused by the loss of dopaminergic neurons in people with PD.

A number of studies using several models, including neuronal and microglial cell lines as well as MPTP-induced mouse and 6-OHDA rat models, have demonstrated that imatinib and other TKIs protect dopaminergic cells against degeneration and produce additional antiparkinsonian effects [2,10,20,21,22,23,24,25]. These activities of TKIs are not surprising, since there is an increase in the level and activity of c-Abl in mostly PD-affected areas of the brain, such as the substantia nigra and the striatum [22,26,27,28]. This abnormal c-Abl function is triggered by oxidative stress, which is a characteristic feature of both sporadic and familial PDs. There is experimental evidence that, by reducing the aberrant over-activation of c-Abl, TKIs can restore the function of key proteins such as parkin [27,29,30], α-synuclein, p-p38 alpha, and NF-κB, whose activation in response to oxidative stress leads to toxicity or neuronal death [22,31,32].

Also, this study indicated that imatinib reduced the oxidative stress (a reduction of 26% at the highest concentration—5 μM) induced by MPP^+^, which is the gold standard in PD scientific research. The conjugate (imatinib+TP10), with the linker or without, produced a more evident effect in this respect, with a concentration-dependent inhibition oscillating between 61 and 64%. Considering both CPPs used in this study, TP10 did not affect the post-MPP^+^ oxidative stress and TAT even enhanced it. Moreover, contrary to imatinib+TP10, the conjugate of imatinib and TAT intensified the response due to the MPP^+^ treatment (ca. 39%). This unexpected feature has led to the discontinuation of further research into imatinib+TAT. Hence, it appears that not all CPPs share the favorable features of TP10, which could be utilized for imatinib delivery into cells. 

It is known that the exposure of neurocytes to oxidative stress may bring about their annihilation. Under PD conditions evoked by MPP^+^, apoptosis is one of the main mechanisms of programmed cell death. This neurotoxin directs the cell to the inner pathway of apoptosis, which depends on the dysfunction of mitochondria and proteolytic enzymes—caspases [33]. 

Accordingly, any circumstances leading to the inhibition of oxidative stress or its pathological consequences will result in diminished apoptosis, which would impede the propagation of neuronal loss in PD. One of the possible strategies that could halt the apoptotic signaling pathway is the inhibition of c-Abl upregulation. The approach utilized in this study indicates that imatinib elicited a slight inhibition of apoptosis, as reflected by the diminished luminescence ranging from 10 to 23%. Similarly, as in the case of oxidative stress, the conjugate more pronouncedly affected MPP^+^-induced apoptosis so that the luminescence was reduced by 35–56%. As could be expected, apoptosis did not change after the TP10 treatment. 

Although MPP^+^ causes neuronal loss mainly by caspase-dependent apoptosis, other mechanisms of death may also participate [33]. It has been found that high doses of MPP^+^ may result in necrotic death due to dramatic cellular decreases in ATP levels [34]. However, that was not the case in this study. In the experimental set presented here, MPP^+^ induced only small increases in the fluorescence, so that the probable significance of necrosis is rather negligible.

Irrespective of the type of death produced by MPP^+^, the conjugate’s neuroprotective activity also became visible in the fluorescence microscopy experiment; the compound was able to restore the number of cells to the level observed before the neurotoxin treatment. 

There are several controversies as to whether an imatinib treatment could be successfully adopted for PD therapy. These controversies concern imatinib’s protection against neurodegeneration and access to the brain [26,27,35]. Despite its lipophilicity, this drug is poorly distributed in the CNS, mostly due to the activation of the efflux transporters ABCB1 and ABCG2 [28,36,37]. The activity of ABCB1 was also an object of this study. As could be expected, imatinib activated this efflux pump. Furthermore, a similar effect was observed after the TP10 treatment. This CPP is known to have activating properties, at least with respect to ABCB1 proteins. Interestingly, there was no impact on them due to imatinib+PEG_4_+TP10 exposure. Evidently, the mixture lost its affinity, at least for the ABCB1 efflux pump. Perhaps this resulted from the interactions (either pharmacokinetic or pharmacodynamic with the resultant synergistic or antagonistic effect) between its components, i.e., imatinib and TP10. Such a phenomenon has been observed with mixtures consisting of TP10 and cisplatin or TP10 and vancomycin [15,16]. 

Considering the direct deleterious impact of the compounds in question on HT-22 cells (expressed by the cytotoxicity assays and calculated IC50 values), it should be emphasized that their inhibitory potential was relatively low at the concentration used in this study (2.5 μM). However, at higher concentrations, the cytotoxicity increases, particularly after TP10. At high concentrations, this CPP induces morphology modifications associated with cell membrane weakness and instability [38]. Interestingly, its cytotoxicity does not affect that of the construct. As the performed tests indicated, its cytotoxic effect was comparable to that of imatinib itself. Perhaps the non-covalent interactions between the molecule of imatinib and that of TP10 occurring during the mixing process bring about the improved safety of imatinib+PEG_4_+TP10. 

In conclusion, the avoidance of one of the efflux pumps by imatinib+PEG_4_+TP10 may explain its better access to the cell interior in comparison to that of imatinib itself. This quality, in turn, may account for the superior activity of the conjugate with respect to the inhibition of MPP^+^-induced oxidative stress and apoptosis as well as the decreased mortality of the tested cells. 

At present, it is difficult to predict whether the lack of activation of the ABCB1 system found in this study may also be ascribed to that present in the BBB. Theoretically, if that were the case, it would translate into a better distribution of imatinib (in the form of a conjugate with TP10) within the CNS, and by this means, the drug could produce a more prominent neuroprotective effect. Additionally, it should be emphasized that the direct cytotoxicity of the conjugate was rather small.

Needless to say, this study presents preliminary research. However, its results may be an incentive for further investigations aiming at developing TKI-based therapeutic interventions for PD in the future. 

## Data Availability

Rusiecka, Izabela, 2024; raw results from the MTT assay obtained after incubation of HT-22 neuronal cells; https://ppm.gumed.edu.pl/info/researchdata/GUMfc683cc0f47d44f2bd19df66cf8d0403/ (accessed on 26 April 2024).

## Supplementary Materials

The following supporting information can be downloaded at: https://www.mdpi.com/article/10.3390/pharmaceutics16060778/s1, Figure S1: HPLC analysis of TP10; Figure S2: HPLC analysis of TAT.
