# Supplementary material for: Neuroprotective Activity of a Non-Covalent Imatinib+TP10 Conjugate in HT-22 Neuronal Cells In Vitro
Source: Pharmaceutics. 2024 Jun 7;16(6):778. doi: 10.3390/pharmaceutics16060778 (PMC11207969; doi:10.3390/pharmaceutics16060778)
Supplement: Supplementary file 1 [file pharmaceutics-16-00778-s001.zip › Fig. S1.pdf]

pepMic
COMMITTED TO LIFE SCIENCE

HPLC Analysis

Name	:TP10
Sequence	:AGYLLGKINLKALAALAKKIL-NH2
Lot.No	:PCM14815-1118
Pump A	:0.1%Trifluoroacetic in 100% water
Pump B	:0.1%Trifluoroacetic in 100% acetonitrile
Total Flow	:1ml/min
Wavelength	:220nm
Analytical column type	:SHIMADZU Inertsil ODS-SP(4.6*250mm*5um)
Dissolution method	:15%ACN+85%H2O
Inj. Volume	:12 uL

Time	Module	Action	Value
0.01	Pumps	B.Conc	25
20.00	Pumps	B.Conc	65
30.00	Pumps	B.Conc	100
38.00	Pumps	B.Conc	100
40.00	Pumps	B.Conc	25
50.00	Controller	Stop	

Chromatogram

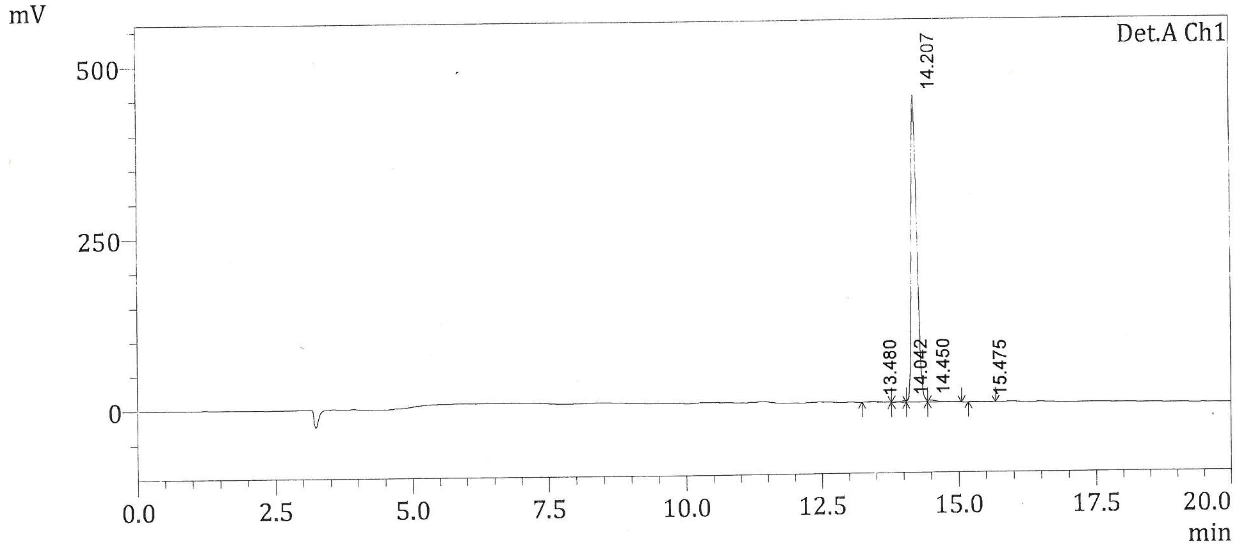

1 Det.A Ch1/220nm

PeakTable

Detector A Ch1 220nm

Peak#	Ret. Time	Area	Height	Area %	Height %
1	13.480	22246	1445	0.661	0.317
2	14.042	18631	2287	0.553	0.502
3	14.207	3269614	447702	97.088	98.202
4	14.450	40083	3381	1.190	0.742
5	15.475	17100	1084	0.508	0.238
Total		3367675	455900	100.000	100.000

Pepmic Co.,Ltd

Tel: +86-512-65834896 Email: info@pepmic.com Web: www.pepmic.com

Address: 35 Xingxian Road, High-tech Development Zone, Suzhou, China 215151

MS Spectrum Graph

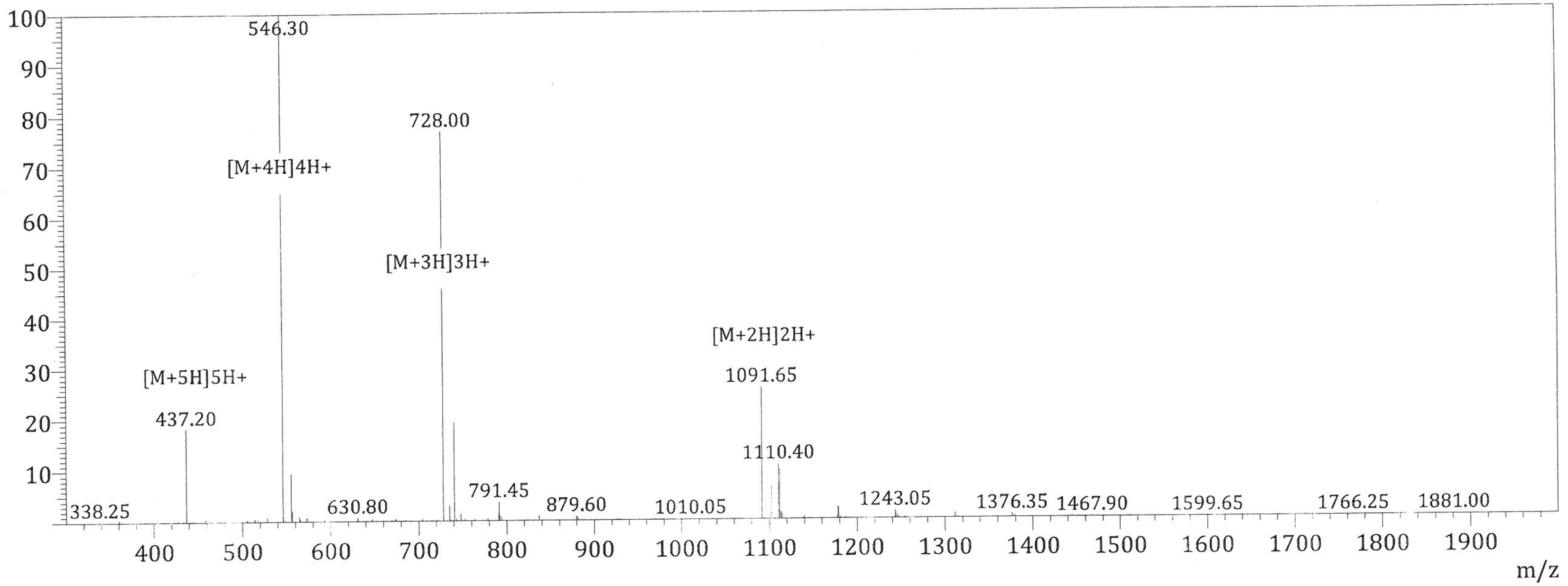

Sample Information

Dissolution method	:5%HAC+8%ACN+87%H2O	Interface	:ESI	Prerod Bias	:+1.5kv
Modified Date	:2019/11/27	Nebulizing Gas Flow	:1.50L/min	Detector	:-0.2kv
Injection Volume	:1ul	CDL Temp	:250C	T.Flow	:0.2ml/min
Heat Block Temp	:200	CDL Volt	:0v	B.conc	:50%H2O/50%MEOH

Name	:TP10
Sequence	:AGYLLGKINLKALAALAKKIL-NH2
Lot.No	:PCM14815-1118
Theoretical	:2181.73
Observed	:2181.20

Pepmic Co.,Ltd
Tel: +86-512-65834896 Email: info@pepmic.com Web: www.pepmic.com
Address: 35 Xingxian Road, High-tech Development Zone, Suzhou, China 215151
